# Folate receptor-positive circulating tumor cells predict survival and recurrence patterns in patients undergoing resection for pancreatic cancer

**DOI:** 10.3389/fonc.2022.1012609

**Published:** 2022-10-13

**Authors:** Hao Cheng, Jun Yang, Xu Fu, Liang Mao, Xuehui Chu, Chenglin Lu, Gang Li, Yudong Qiu, Wei He

**Affiliations:** ^1^ Department of General Surgery, Nanjing Drum Tower Hospital, The Affiliated Hospital of Nanjing University Medical School, Nanjing, China; ^2^ Department of Pathology, Nanjing Drum Tower Hospital, The Affiliated Hospital of Nanjing University Medical School, Nanjing, China; ^3^ Institute of Thoracic Oncology, West China Hospital, Sichuan University, Chengdu, China

**Keywords:** pancreatic cancer, folate receptor, circulating tumor cells, surgical resection, prognosis, recurrence

## Abstract

**Objective:**

To evaluate the prognostic impact of folate receptor (FR)-positive circulating tumor cells (FR^+^ CTCs) for patients with pancreatic cancer (PC).

**Background:**

Risk stratification before surgery for PC patients remains challenging as there are no reliable prognostic markers currently. FR^+^ CTCs, detected by ligand-targeted polymerase chain reaction (LT-PCR), have shown excellent diagnostic value for PC in our previous study and prognostic value in a variety of cancer types.

**Methods:**

Peripheral blood samples from 44 consecutive patients diagnosed with PC were analyzed for FR^+^ CTCs. 25 patients underwent tumor resection and were assigned to the surgical group. 19 patients failed to undergo radical resection because of local advance or distant metastasis and were assigned to the non-surgical group. The impact of CTCs on relapse and survival were explored.

**Results:**

For the prognostic stratification, the optimal cut-off value of CTCs analyzed by receiver operating characteristic (ROC) curve was 14.49 folate units (FU)/3 ml. High CTC levels (> 14.49 FU/3 ml) were detected in 52.0% (13/25) of the patients in the surgical group and 63.2% (12/19) in the non-surgical group. In the surgical group, median disease-free survival (DFS) for patients with high CTC levels versus low CTC levels (< 14.49 FU/3 ml) was 8.0 versus 26.0 months (*P* = 0.008). In multivariable analysis, CTCs were an independent risk factor for DFS (HR: 4.589, *P* = 0.012). Concerning the recurrence patterns, patients with high CTC levels showed a significantly frequent rate of distant and early recurrence (*P* = 0.017 and *P* = 0.011). CTC levels remained an independent predictor for both distant (OR: 8.375, *P* = 0.014) and early recurrence (OR: 8.412, *P* = 0.013) confirmed by multivariable logistic regression. However, CTCs did not predict survival in the non-surgical group (*P* = 0.220).

**Conclusion:**

FR^+^ CTCs in resected PC patients could predict impaired survival and recurrence patterns after surgery. Preoperative CTC levels detected by LT-PCR may help guide treatment strategies and further studies in a larger cohort are warranted.

## Introduction

Pancreatic cancer (PC) is the fourth leading cause of cancer mortality in the United States, with an estimated 62,210 new cases diagnosed in 2022 (1). It is currently predicted to become the second leading cause by 2030 (2). Radical resection, in combination with systemic therapy, remains the only hope of cure or meaningful long-time survival with overall 5-year survival rates as high as 30% for patients with PC (3). Due to a combination of late presentation and early metastasis, 80% of the patients are diagnosed in the advanced stage and only 10%-20% of PC patients can get a curative resection at the time of diagnosis. However, due to a substantial rate of under-staging, around 20% of patients already recure within the first 6 months after surgery, resulting the median disease-free survival (DFS) is just over 12 months (4, 5). Therefore for many of these patients, the survival advantage from curative resection is questionable. The main cause of early relapse is likely occult systemic disease below the detection limit of cross-sectional imaging, although there may be other contributing factors such as postoperative morbidity (6, 7). Neoadjuvant therapy is recently recommended to address the issue of occult systemic disease (8). Currently, selection of potentially resectable patients for surgery remains challenging as there are no methods to stratify a patient’s risk for metastasis to help guide neoadjuvant and adjuvant therapies (9). One possible strategy is to identify effective prognostic biomarkers to distinguish patients at high risk of early systemic progression who may benefit from systemic treatment first and patients with favourable prognosis who are more likely to benefit from upfront resection (10).

Circulating tumor cells (CTCs), functioning as the “seeds” of metastasis, are tumor cells that originate from primary tumors, survive in circulating and disseminate to colonize distant sites through invading adjacent vasculature (11, 12). Involvement of CTCs in the metastatic process has been identified in the majority of solid tumors (13–15). Accumulating evidence has demonstrated that, as a non-invasive assessment of tumor biology, CTCs are a readily available biomarker for predicting survival in colorectal, breast, and prostate cancers (16–18). In patients with potentially resectable PC, the lack of reliable biomarkers to guide surgical or neoadjuvant treatment decisions also motivated the present studies to investigate the prognostic impact of preoperative CTCs analysis (19–22). Thus far, the CellSearch System remains the first and only CTC detection platform approved by Food and Drug Administration (FDA), although detection, enumeration, and isolation of CTCs have been facilitated by recent technological advancements in a sensitive and reproducible manner for clinical and research applications. It utilizes immunomagnetic separation for isolation of CTCs, and then quantitative evaluation of CTCs is analyzed by capturing epithelial cell adhesion molecule (EpCAM), an epithelial cell marker (23). CellSearch is also the most commonly used method to examine CTCs as a prognostic marker in PC patients in a number of studies, however, across various stages of PC, it has performed with relatively poor detection rates of 7–48% (24–26). In addition, in a study evaluating the efficiency of the CellSearch System, CTC detection rate and counts have been found to be lowest in PC among different types of metastatic cancers (27). Therefore, diverse techniques and technologies have been developed for enrichment, isolation, and identification of CTCs from peripheral blood samples in PC patients in recent years.

Folate receptors (FRs), cysteine-rich cell-surface glycoproteins, are highly expressed in a variety of cancers, including PC (28–31). Our previous study has shown promising clinical value of detecting FR-positive (FR^+^) CTCs by a novel ligand-targeted polymerase chain reaction (LT-PCR) method in patients with PC (32). Although FR^+^ CTCs have been demonstrated to be useful in the diagnosis of PC, their application in predicting prognosis requires further clarification. Therefore, the purpose of this study was to investigate the prognostic impact of pretreatment FR^+^ CTCs analysis in patients with PC.

## Patients and methods

### Patients, study design, and clinical data collection

Between September 2018 to December 2019, 50 consecutive patients with suspected PC treated at our hospital were enrolled into this observational study. The treatment strategies for all patients were discussed and determined by the multidisciplinary team of pancreatic disease, independent of the results of CTCs analysis. Exclusion criteria were (1): a history of any other malignancy or any anticancer therapies in the last 10 years (2); failure to adhere to standard surgical procedures in the surgical group (3); other than PC confirmed by final histopathologic observations. Diagnosis was confirmed from the specimens obtained by resection or biopsy. A database of demographic, laboratory, and relevant clinicopathologic variables, including preoperative carbohydrate antigen 19-9 (CA19-9), age, gender, location of primary tumor, tumor differentiation, tumor size, tumor stages et al, was prospectively maintained. Disease stages - tumor, node, and metastasis (TNM) staging, were based on the eighth edition of the American Joint Committee on Cancer (AJCC) manual. A distance from the tumor to the resection margin≥1 mm was defined as R0.

Standard pancreaticoduodenectomy (PD), distal pancreatectomy (DP) or other procedures were performed in patients who were selected for radical surgery in accordance with the tumor location and extension. In addition, patients were followed with a standard postoperative protocol, with routine postoperative clinical status and CA19-9 assessment every 3 months and contrast-enhanced computerized tomography (CT) or magnetic resonance imaging (MRI) scan every 6 months. If necessary, positron emission tomography (PET) was conducted to evaluate recurrence. Early recurrence was defined as within 12 months of surgery, as described in previous studies (5, 33). Locoregional recurrence was defined as recurrent disease along the superior mesenteric artery (SMA)/superior mesenteric vein (SMV), celiac axis, or porta hepatis and in the pancreatic bed, retroperitoneum, or remnant pancreas through radiographic or pathological evidence. Distant recurrence was tumor spread outside of the locoregional area (liver, lungs, peritoneum and extra-regional lymph nodes) (34). All procedures were in accordance with the ethical standards of the Helsinki Declaration. The study was approved by the Ethics Committee of Nanjing Drum Tower Hospital (No. 2020-079-01), and informed written consent was obtained from all subjects before the study.

### CTC detection

Before commencing treatment, peripheral venous blood samples (3 ml) from every patient were collected in vacuum tubes containing the anticoagulant ethylenediaminetetraacetic acid for CTCs analysis. For patients with neoadjuvant treatment followed by curative-intent pancreatectomy, CTC detection was performed before surgery. A commercially available CTC detection kit, CytoploRare Kit, invented by Geno Biotech and approved by the China FDA, was used for isolation, enrichment and enumeration of FR^+^ CTCs, as described and detailed previously (32). All blood specimens were stored at 4°C and processed within 24 hours of blood withdrawal. The experimental procedure was performed strictly according to the manufacturer’s protocol. Briefly, the isolation and enrichment of CTCs was initially achieved by lysing erythrocytes, followed by immunomagnetic depletion of leukocytes from the whole blood. Then, LT-PCR was used for quantitative analysis of the FR^+^ CTCs in each blood sample. Finally, the level of FR^+^ CTCs in each sample was calculated on the basis of a calibration curve generated with the standard reference materials provided in the kit. FR^+^ CTC levels were measured in folate units (FU)/3 ml of blood.

### Statistical analysis

Continuous variables were presented as medians (ranges) and categorical variables were summarized as number (percentage). Statistical analysis for these two types of variable was examined using Student’s t test (or Mann-Whitney U test) and Fisher’s exact test, respectively. Optimal cut-off value of CTCs for recurrence prediction was analyzed by receiver operating characteristic (ROC) curve and calculated with Youden Index. Survival analyses were carried out with the Kaplan-Meier method, using Log-rank test for difference of curve pairs. Overall survival (OS) was defined as the time from date of diagnosis or resection to either death by any cause or censored at last follow-up. DFS was defined as the time interval between date of operation and date of tumor relapse showed by radiological or clinical evidence. To assess the independent influence of CTCs and other covariates on tumor recurrence, univariable and multivariable Cox Proportional Hazard regression model analyses were performed. Multivariable analyses for distant and early recurrence were calculated with a logistic regression model. Variables with a *P* value < 0.2 in univariable analyses and clinically relevant variables were included in multivariable analyses. Data were analyzed using IBM SPSS, v.25 (IBM Corp., Armonk, NY) and graphs were prepared using PRISM 8 (GraphPad Software, Inc, La Jolla, CA). A *P* value < 0.05 was considered statistically significant.

## Results

### Patient characteristics

**Figure 1:**
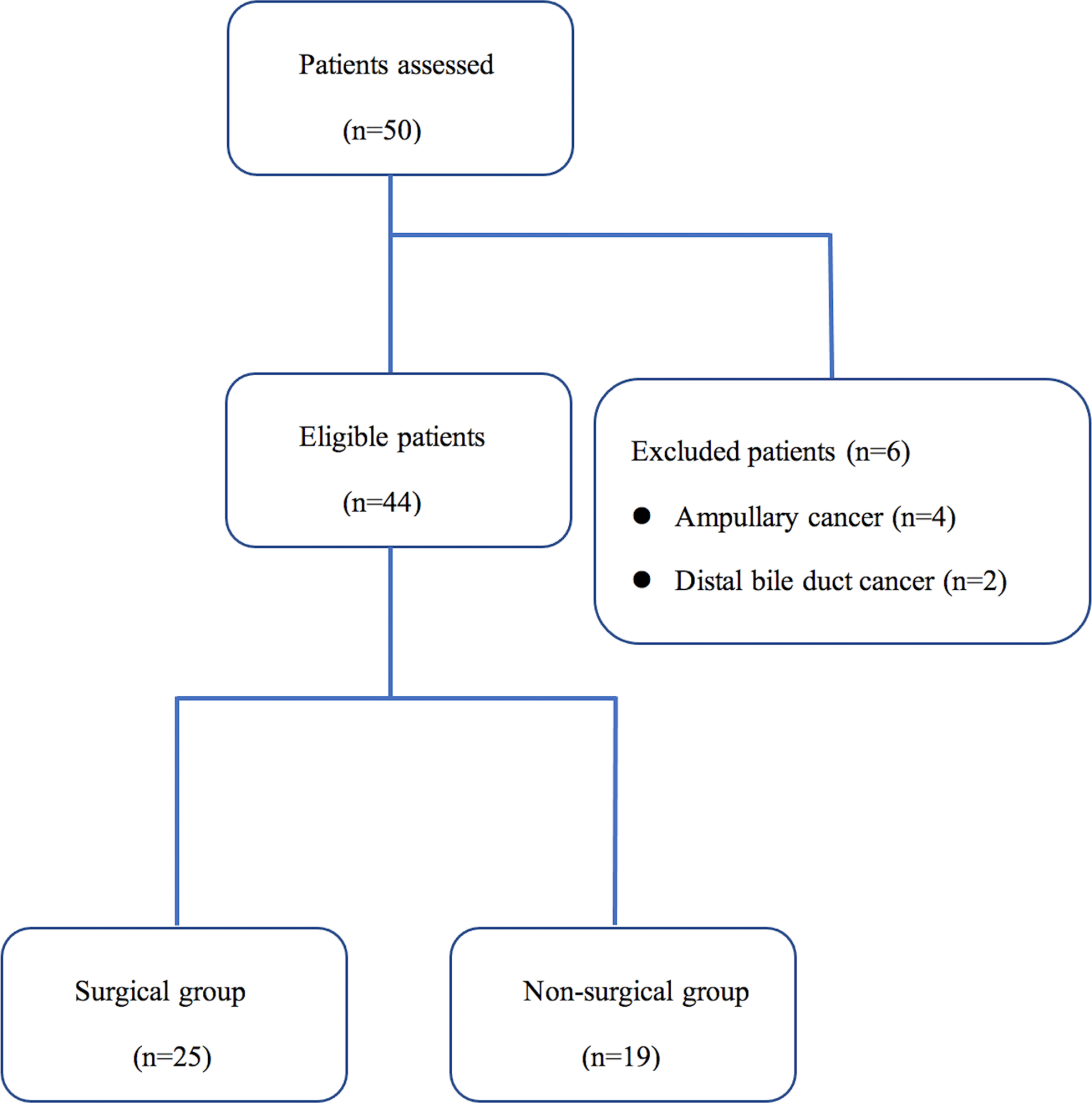
Flowchart of study cohort into 2 groups with different treatment strategies.

Of the 50 patients assessed during the study period, 6 patients (12.0%) did not meet the inclusion criteria. The Consort diagram showing the stratification of the 44 eligible cases is presented in **Figure 1**. Tumor resection with curative intent was performed in 56.8% of the patients (25/44) (surgical group), while 43.2% (19/44) were not resected due to local advance or distant metastasis (non-surgical group) (**Table 1**). The median age of these enrolled patients was both 64 years in the two groups and 60.0% (15/25) were men in the surgical group, while 57.9% (11/19) in the non-surgical group. In the surgical group, 60.0% of the tumors were located in the head, and according to the histopathologic type, most were pancreatic ductal adenocarcinoma (PDAC), and only two were malignant intraductal papillary mucinous neoplasm (IPMN). R0 resection was achieved in 56.0% (14/25) of the patients and 80.0% (20/25) received adjuvant treatment. 3 patients received neoadjuvant chemotherapy preoperatively according to the decision taken on pancreatic multidisciplinary and the regimen was gemcitabine with albumin-bound paclitaxel for all patients. In the non-surgical group, 73.7% (14/19) of the patients received systemic treatment.

**Table 1:** Demographics, clinicopathologic, and treatment characteristics of included patients.

Characteristics	Surgical group (n=25)	Non-surgical group (n=19)
Age, years median (range)	64 (45–87)	64 (42-79)
Sex, male n (%)	15 (60.0)	11 (57.9)
Preoperative CA19-9 level U/ml, n (%)		
≥ 200	9 (36.0)	12 (63.2)
< 200	16 (64.0)	7 (36.8)
Operation type, n (%)		
PD	14 (56.0)	BDB 9 (47.4)
DP	10 (40.0)	EUS-FNA 10 (52.6)
TP	1 (4.0)	
Venous resection, n (%)	3 (12.0)	n.a.
Histopathologic type, n (%)		
PDAC	23 (92.0)	19 (100.0)
Malignant IPMN	2 (8.0)	0 (0.0)
Tumor size, mm median (range)	30 (12-95)	n.a.
Tumor location, n (%)		
Head	15 (60.0)	11 (57.9)
Body or tail	10 (40.0)	8 (42.1)
Tumor stage, n (%)		
I	9 (36.0)	0 (0.0)
II	11 (44.0)	0 (0.0)
III	5 (20.0)	11 (57.9)
IV	0 (0.0)	8 (42.1)
Tumor differentiation, n (%)		
Well	4 (16.0)	3 (15.8)
Moderate	10 (40.0)	5 (26.3)
Poor	11 (44.0)	1 (5.3)
Not specified	0 (0.0)	10 (52.6)
Resection margin, n (%)		
R0	14 (56.0)	n.a.
R1	11 (44.0)	n.a.
Adjuvant treatment/Systemic treatment, n (%)		
Yes	20 (80.0)	14 (73.7)
No	5 (20.0)	5 (26.3)
Neoadjuvant treatment, n (%)		
Yes	3 (12.0)	n.a.
No	22 (88.0)	n.a.

BDB, biliodigestive bypass; CA19-9, carbohydrate antigen 19-9; DP, distal pancreatectomy; EUS-FNA, endoscopic ultrasound guided fine needle aspiration; IPMN, intraductal papillary mucinous neoplasm; n.a, not applicable; PD, pancreaticoduodenectomy; PDAC, pancreatic ductal adenocarcinoma; TP, Total pancreatectomy.

### CTC levels in the patient cohort

**Figure 2:**
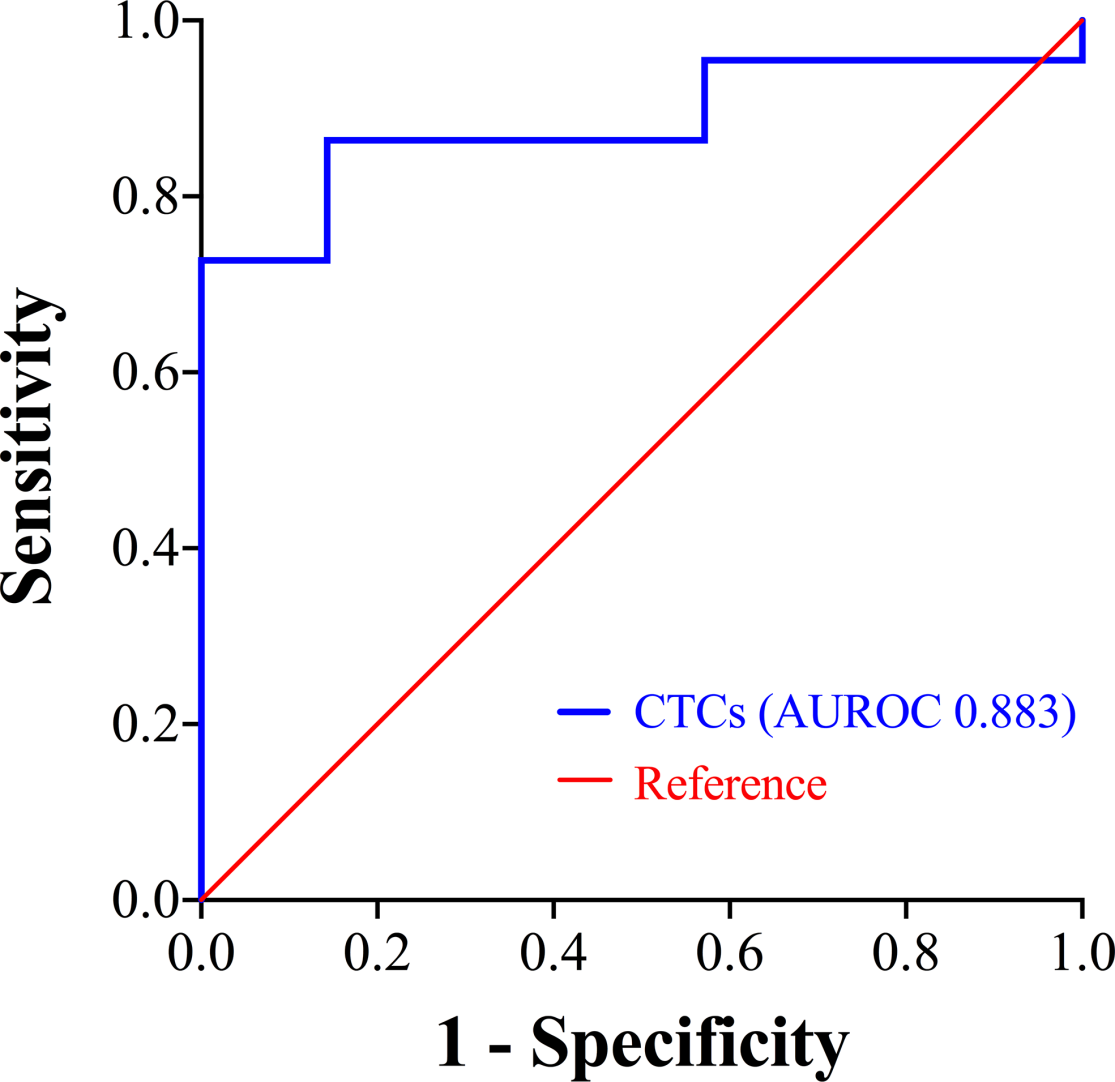
ROC curve showing the prognostic stratification performance of CTCs. CTCs, circulating tumor cells; ROC, receiver operating characteristic; AUROC, area under the ROC curve.

For the prognostic stratification, ROC curve analysis showed that the area under the ROC curve (AUROC) was 0.883 (*P* = 0.003), with 14.49 FU/3 ml as the optimal cut-off value of CTCs and the Youden Index was 0.727 (**Figure 2**). The CTC levels of patients in the surgical group (median 14.92 FU/3 ml, range 5.61 to 26.98 FU/3 ml) were slightly lower than those of patients in the non-surgical group (median 16.76 FU/3 ml, range 5.49 to 41.22 FU/3 ml), although the difference was not significant (*P* = 0.147). In the surgical group, high CTC levels (> 14.49 FU/3 ml) were detected in 52.0% (13/25) of the patients and in the non-surgical group, 12 patients had high CTC levels. We also compared the clinicopathological characteristics of patients with high and low CTC levels, and the results are shown in **Table 2**. In the surgical group and non-surgical group, there were both no significant difference of clinicopathological characteristics between the two subgroups.

**Table 2:** Comparison of the clinicopathological characteristics of patients with high and low CTC levels in different groups.

Characteristics	Surgical group (n=25)			Non-surgical group (n=19)		
	High CTC levels(n=13)	Low CTC levels(n=12)	*P*	High CTC levels(n=12)	Low CTC levels(n=7)	*P*
Age, years median (range)	65 (55-81)	64 (45-87)	0.970	63.5 (42-79)	63 (58-77)	0.590
Sex, male n (%)	8 (61.5)	7 (58.3)	1.000	6 (50.0)	5 (71.4)	0.633
Preoperative CA19-9 level U/ml, n (%)			0.688			1.000
≥ 200	4 (30.8)	5 (41.7)		8 (66.7)	4 (57.1)	
< 200	9 (69.2)	7 (58.3)		4 (33.3)	3 (42.9)	
Tumor size, mm median (range)	27.0 (12-95)	37.5 (20-61)	0.164	n.a.		
Tumor location, n (%)			0.111			0.960
Head	10 (76.9)	5 (41.7)		7 (58.3)	4 (57.1)	
Body or tail	3 (23.1)	7 (58.3)		5 (41.7)	3 (42.9)	
Tumor stage, n (%)			0.645			
I+II	11 (84.6)	9 (75.0)		0 (0.0)	0 (0.0)	
III+IV	2 (15.4))	3 (25.0)		12 (100.0)	7 (100.0)	
Tumor differentiation, n (%)			0.428			0.342
Well+Moderate	6 (46.2)	8 (66.7)		6 (50.0)	2 (28.6)	
Poor	7 (53.8)	4 (33.3)		1 (8.3)	0 (0.0)	
Not specified	0 (0.0)	0 (0.0)		5 (41.7)	5 (71.4)	
Resection margin, n (%)			1.000		
R0	7 (53.8)	7 (58.3)		n.a.		
R1	6 (46.2)	5 (41.7)		n.a.		
Adjuvant treatment/Systemic treatment, n (%)			0.645			0.603
Yes	11 (84.6)	9 (75.0)		8 (66.7)	6 (85.7)	
No	2 (15.4)	3 (25.0)		4 (33.3)	1 (14.3)	

CA19-9, carbohydrate antigen 19-9; n.a, not applicable.

### CTC detection and survival

**Figure 3:**
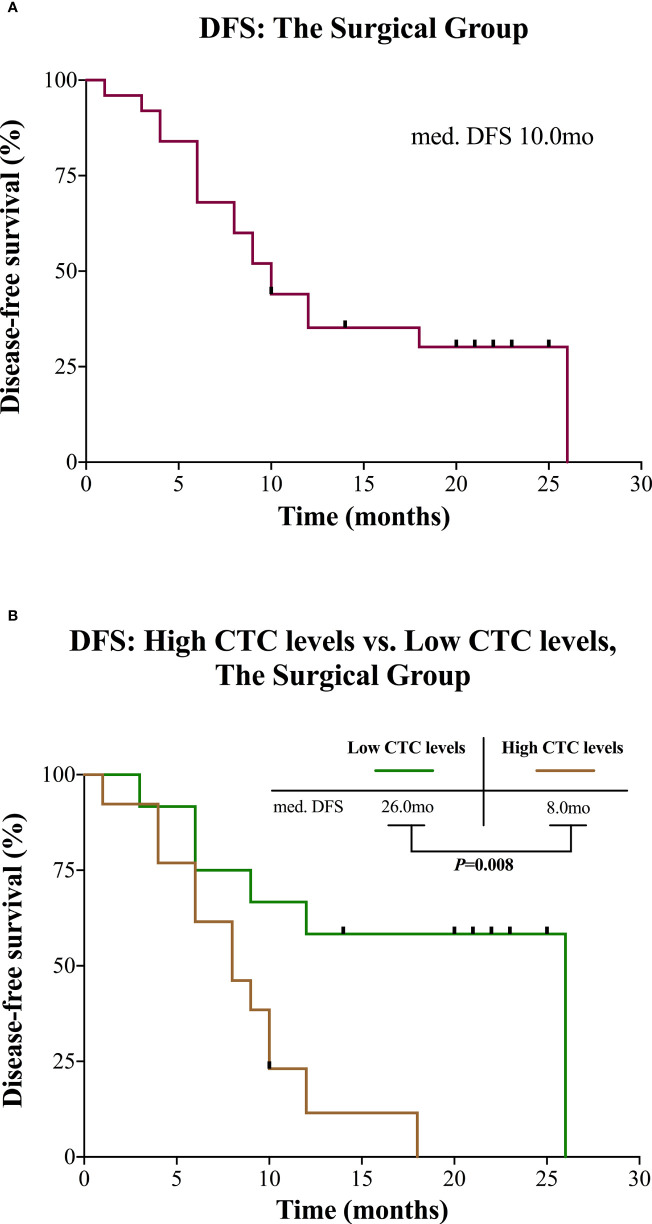
Kaplan-Meier curves for patients in the surgical group. **(A)** DFS for the entire group. **(B)** DFS of the surgical group divided into high and low CTC levels. CTC, circulating tumor cell; DFS, disease-free survival; mo, months; med., median.

In the surgical group, the median observation time was 20.0 months (range 6.0 to 28.0). Median DFS was 10.0 months for all patients (**Figure 3A**). Patients with high CTC levels had a significantly shorter DFS (median 8.0 vs. 26.0 months, *P* = 0.008; **Figure 3B**) compared with patients with low CTC levels. The univariable survival analyses of DFS for CTC levels (HR 3.735; 95% CI: 1.263–11.049; *P* = 0.017) and other risk factors are presented in **Table 3**. Of those, age also affected the prognosis. After inclusion of these confounding factors as well as other known risk factors (R, tumor stage) in multivariable analysis, CTC levels remained a significant prognostic factor (HR 4.589; 95% CI: 1.404–14.997; *P* = 0.012; **Table 3**).

**Table 3:** Uni- and multivariable analyses of prognostic factors of DFS for patients in the surgical group.

Variable	Univariable		Multivariable	
	HR (95% CI)	*P*	HR (95% CI)	*P*
Sex, female vs. male	0.530 (0.185-1.514)	0.236		
Age, years	**1.038 (0.992-1.086)**	**0.110**	1.054 (0.999-1.113)	0.054
CA19-9, U/ml	1.001 (0.999-1.002)	0.342		
Tumor site, Body/tail vs. Head	0.814 (0.298-2.223)	0.688		
Neoadjuvant treatment, Yes vs. No	0.389 (0.052-2.940)	0.360		
Adjuvant treatment, Yes vs. No	0.850 (0.276-2.616)	0.777		
R, R1vs. R0	1.074 (0.414-2.787)	0.883	0.733 (0.252-2.128)	0.568
CTC levels, High vs. Low	**3.735 (1.263-11.049)**	**0.017**	**4.589 (1.404-14.997)**	**0.012**
Tumor stage	1.302 (0.652-2.602)	0.454	1.867 (0.922-3.781)	0.083

Age and CA19-9 were calculated as continuous variables. CTC, circulating tumor cell; CA19-9, carbohydrate antigen 19-9; CI, Confidence interval; DFS, disease-free survival; HR, Hazard ratio.

Shown in bold are univariable associations (P < 0.2) that were selected for multivariable analysis and significant risk factors (P < 0.05) on multivariable analysis.

**Figure 4:**
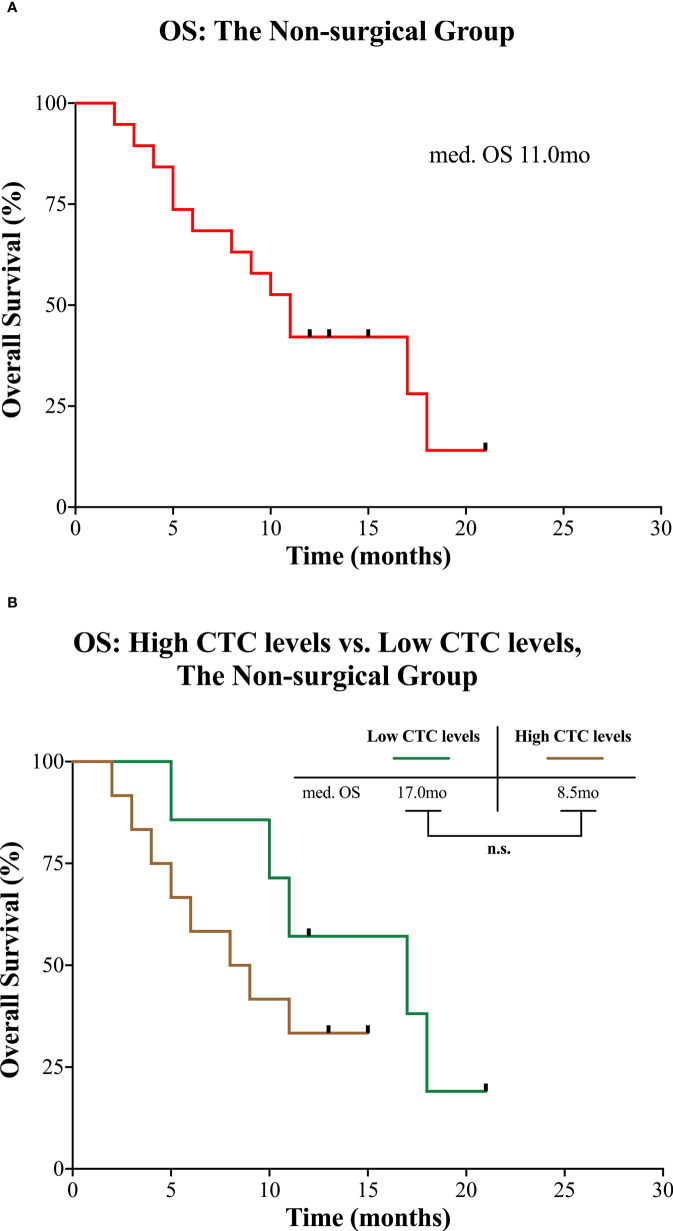
Kaplan-Meier curves for patients in the non-surgical group. **(A)** OS for the entire group. **(B)** OS of the non-surgical group divided into high and low CTC levels. CTC, circulating tumor cell; mo, months; med., median; n.s., not significant; OS, overall survival.

In the non-surgical group, the median observation time was 13.0 months (range 2.0 to 21.0). Median OS was 11.0 months for all patients (**Figure 4A**). Median OS for patients with high CTC levels was 8.5 months and 17.0 months for patients with low CTC levels, but the difference was not statistically significant (*P* = 0.220; **Figure 4B**).

### Patterns of recurrence according to the CTC levels in the surgical group

**Figure 5:**
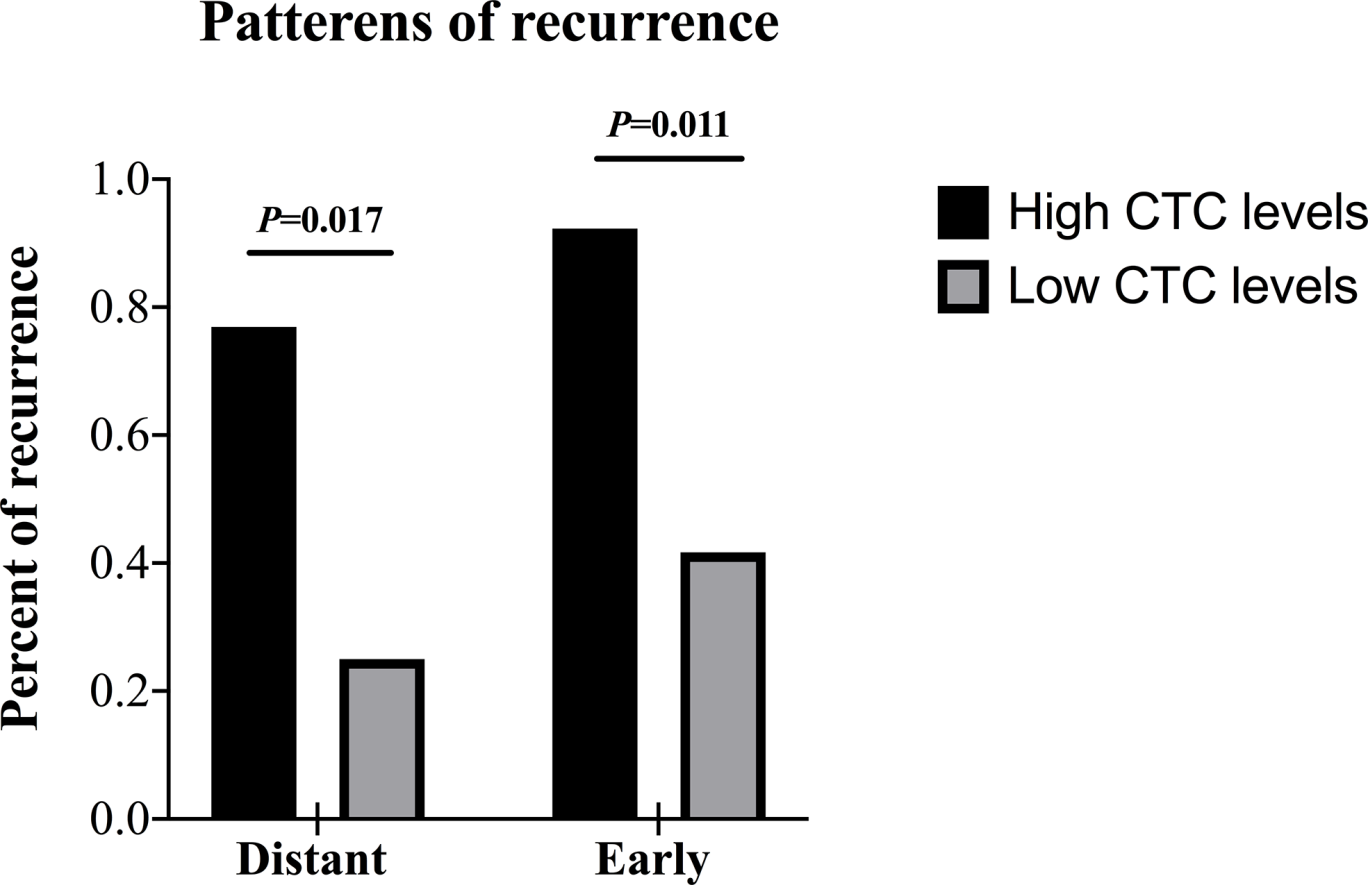
Patterns of recurrence of patients in the surgical group according to the CTC levels. CTC, circulating tumor cell.

We compared the presence or absence of recurrence in accordance with CTC levels, and we found that recurrence was significantly higher in the patients with high CTC levels (12/13, 92.3%) compared with patients with low CTC levels (6/12, 50.0%) (*P* = 0.030). Then the recurrence site and time of emergence were further analyzed (**Table 4** and **Figure 5**). Distant recurrence was significantly higher in the patients with high CTC levels as compared with patients with low CTC levels (76.9% vs. 25.0%, *P* = 0.017). In the patients with low CTC levels, 5 out of 12 (41.7%) instances of recurrence were within 12 months, but nearly all recurrences (12/13, 92.3%) in the patients with high CTC levels occurred within 12 months (*P* = 0.011). Finally, we confirmed using multivariable logistic regression analysis that CTCs are an independent risk factor for both distant (OR 8.375; 95% CI 1.915–28.222; *P* = 0.014) and early recurrence (OR 8.412; 95% CI 2.342–27.234; *P* = 0.013) (**Table 5**).

**Table 4:** Subanalysis of the recurrence patterns according to the CTC levels.

Variable	Total number (%) or median	High CTC levels (n=13)	Low CTC levels (n=12)	*P*
Recurrence				**0.030**
No	7 (28.0%)	1 (7.7%)	6 (50.0%)	
Yes	18 (72.0%)	12 (92.3%)	6 (50.0%)	
Recurrence site				
Distant	13 (52.0%)	10 (76.9%)	3 (25.0%)	**0.017**
Locoregional	5 (20.0%)	2 (15.4%)	3 (25.0%)	
Recurrence time				
≤ 12 months	17 (68.0%)	12 (92.3%)	5 (41.7%)	**0.011**
> 12 months or recurrence (-)	8 (32.0%)	1 (7.7%)	7 (58.3%)	
Follow-up duration (months)				
Median	20.0	15.0	24.0	0.050
Range	6.0-28.0	10.0-21.0	6.0-28.0	

CTC, circulating tumor cell.

Bold values was used for P values < 0.05.

**Table 5:** Uni- and multivariable logistic regression analysis for risk factors in distant and early recurrence.

Variable	Distant recurrence					
	Univariable			Multivariable		
	OR (95% CI)		*P*	OR (95% CI)	*P*		
Sex, female vs. male	0.444 (0.087-2.276)		0.330			
Age, years	0.994 (0.915-1.081)		0.896			
CA19-9, U/ml	1.000 (0.997-1.003)		0.957			
Tumor site, Body/tail vs. Head	0.875 (0.176-4.341)		0.870			
Neoadjuvant treatment, Yes vs. No	0.417 (0.033-5.299)		0.500			
Adjuvant treatment, Yes vs. No	0.667 (0.091-4.889)		0.690			
R, R1vs. R0	**0.317 (0.061-1.644)**		**0.171**	0.121 (0.011-1.370)	0.088		
CTC levels, High vs. Low	**5.000 (1.594-26.732)**		**0.014**	**8.375 (1.915-28.222)**	**0.014**		
Tumor stage	1.024 (0.350-2.997)		0.965	2.058 (0.445-9.525)	0.356		
**Variable**	**Early recurrence**					
	**Univariable**			**Multivariable**			
	**OR (95% CI)**		***P***	**OR (95% CI)**	***P***			
Sex, female vs. male	0.545 (0.099-3.004)		0.486			
Age, years	**1.141 (0.987-1.319)**		**0.074**	1.224 (0.995-1.504)		0.055
CA199, U/ml	1.001 (0.998-1.004)		0.600			
Tumor site, Body/tail vs. Head	0.545 (0.099-3.004)		0.486			
Neoadjuvant treatment, Yes vs. No	0.933 (0.072-12.015)		0.958			
Adjuvant treatment, Yes vs. No	0.464 (0.043-4.997)		0.527		
R, R1vs. R0	1.481 (0.265-8.267)		0.654	1.681 (0.128-22.061)		0.692
CTC levels, High vs. Low	**6.800 (1.617-24.519)**		**0.018**	**8.412 (2.342-27.234)**		**0.013**
Tumor stage	0.908 (0.288-2.861)		0.870	3.124 (0.361-27.002)		0.301

Age and CA19-9 were calculated as continuous variables. CTC, circulating tumor cell; CA19-9, carbohydrate antigen 19-9; CI, Confidence interval.

Shown in bold are univariable associations (P < 0.2) that were selected for multivariable analysis and significant risk factors (P < 0.05) on multivariable analysis.

Based on the results, we summarized the relationship between preoperative CTC levels and postoperative patterns of recurrence. If the CTC levels were high (> 14.49 FU/3 ml) before surgery, it tended to show patterns of early and distant recurrence.

## Discussion

This is the first report to demonstrate the clinical significance of FR^+^ CTCs detected by LT-PCR for predicting the survival in patients with PC. The effect of high CTC levels in the present cohort was detrimental. In patients with resected PC, we demonstrated a strong association between the CTC levels and reduced DFS and in the patterns of recurrence, CTCs were also associated with early and distant recurrence.

As a ‘‘liquid biopsy’’, the utilization of CTCs to assess the tumor biology and guide treatment decisions has developed into an emerging field of study (35). Especially in PC, the current lack of individualized up-front treatment stratification by preoperative risk assessment is a major hindrance for improved treatment results of patients with presumed resectable PC and CTCs have been considered as a very useful biomarker to establish the appropriate therapeutic protocol (22). However, there is a broad heterogeneity in the CTC detection platforms to date and as the “gold standard” technique, the detection rate of the CellSearch system is too low to limit its clinical application (36). In recent years, although the presence of sensitive and reproducible platforms for the isolation, enrichment and detection of CTCs from peripheral blood, methodological standardization for such technologies is still required to have a significant value on clinical care (37). As FRs are also highly expressed in PC, our previous study has shown that FR^+^ CTCs have potential as a biomarker for the diagnosis of PC and LT-PCR is feasible and reliable for detecting FR^+^ CTCs in patients with PC (32). In addition, FR^+^ CTCs have been shown to serve as prognostic markers in several cancer types, including gastric, breast and lung cancers (38–40). In a prospective cohort study including 132 gastric cancer patients, combined model including FR^+^ CTC level and other biomarkers (CA19-9, prealbumin and peripheral lymphocyte count) presented high sensitivity (100%) and moderate specificity (59.3%) in predicting peritoneal metastasis, the preoperative FR^+^ CTC level could also predict short-term recurrence after surgery (38). In another prospective study to investigate the prognostic and predictive significance of FR^+^ CTC in non-small cell lung cancer patients who underwent surgery, patients with lower preoperative CTC level had longer relapse-free survival (RFS) and OS, CTC level (HR = 4.10) and pathological stage (HR = 3.16) were independent prognostic factors of RFS (40). Accordingly, in the present analysis, we gave insight into the association between pretreatment CTC levels and patient survival.

The frequency of CTC-detection varies due to the detection methods and the disease status of the patients (19–22). However, in our current study, with 14.49 FU/3 ml as the optimal cut-off value of CTCs, the detection rate of high CTC levels was 52.0% in the patients with resectable PC and 63.2% in the patients with advanced stage cancer. Compared to previous studies, especially the studies in which Cellsearch was used as the detection method, our study showed a higher detection rate in both early and advanced stage cancer. Consequently, these results revealed again that LT-PCR is feasible and reliable for detecting FR^+^ CTCs in PC patients.

In our cohort, 43.2% of the patients were assigned to the non-surgical group due to local advance or distant metastasis of tumor. In this group, median OS was 11.0 months and there was no significant difference in OS between the patients with high and low CTC levels. These results are consistent with other reports utilizing the different methods (41, 42). However, in our study, we have secured meaningful results for the difference between the two groups (8.5 months vs. 17.0 months) and larger-scale studies are warranted in the future.

In the current study, 56.8% of the patients were assigned to the surgical group and tumor resection with curative intent was performed. Median DFS was 10.0 months for all patients, which was similar to other studies with larger number of patients (5, 34). More importantly, high CTC levels predicted impaired DFS following potentially curative surgery and patients with low CTC levels could have a DFS as long as 26.0 months, which was an amazing result. However, this may be related to the limitations of our study with small number of cases and short duration for follow-up, and only 50.0% of the patients with low CTC levels during the observation time recured. Moreover, multivariable analysis indicated that CTCs are an independent risk factor for DFS. Concerning OS in the surgical group, also due to the short follow-up, we did not analyze the OS.

Although there are abundant studies on the association between CTCs and survival, we are aware of only few reports of an association between CTCs from peripheral blood and recurrence rate or pattern (22, 43). Therefore, in the present analysis, we particularly analyzed whether CTC levels could be used as an indicator of early recurrence and recurrence patterns among these patients. In this cohort, the recurrence was significantly higher in the patients with high CTC levels compared with patients with low CTC levels. Meanwhile, in patients with high CTC levels, early recurrence (i.e. within 12 months post-operatively) and distant recurrence were significantly frequent. Moreover, multivariable logistic regression analysis indicated that CTCs are a risk factor for both early and distant recurrence.

Noteworthy, in the present study, the CTC levels were the only independent predictor of DFS and recurrence patterns although other clinically relevant factors such as preoperative CA19-9 level, R status, neoadjuvant and adjuvant therapy, tumor stage were included in the analyses. However, in some previous studies, including large-scale and multicenter clinical trials, CA19-9 level, R status, neoadjuvant and adjuvant therapy were all significantly associated with survival and recurrence (44–47). The discrepancy may be mainly explained by the small sample size and the selection bias of patients in our study. Another alternate explanation may be that we did not analyze the surgical margins separately as different surgical margins could have different prognostic roles (48).

Our study showed that with the currently available techniques for CTC-detection and treatment modalities in PC, to support surgical treatment decisions probably would be a promising use of CTC-analysis. Similar to CTCs, circulating tumor DNA (ctDNA) also has the potential to be a preoperative prognostic tool for the stratification of patients with resectable PC (49–51). Although ctDNA was more abundant and easier to detect than CTCs in comparative studies, the prognostic impact of different mutational signatures in ctDNA was not fully resolved (52–54). In a recent meta-analysis, ctDNA was detected in 8.3–68.6% of patients with resectable PDAC preoperatively and was associated with lower RFS and OS (49). Most probably, future improvements in systemic treatment are dependent upon identification of the core molecular characteristics or driver mutations of the cancer cells.

We acknowledge that our study has several important limitations. First, the number of patients is relatively small and the follow-up time is relatively short, which limit the generalization of the results reported. In addition, FR^+^ CTCs can not predict survival in the non-surgical group, indicating that this prognostic biomarker has less utility in patients with advanced disease, which might mainly due to the universally poor prognosis of this patient population. Moreover, the present study design did not include the CTC detection at multiple time points to observe how CTC dynamics predict outcome with treatment, however, our study does provided evidence on the utility of FR^+^ CTCs as a biomarker at a time when key treatment decisions are made. Finally, the recurrences of some patients were identified based on radiological evidence, without pathological verification, which may include potential for some provider variability regarding postoperative imaging.

In conclusion, this small-scale, exploratory clinical trial revealed that FR^+^ CTCs detected by LT-PCR predict shorter DFS and are associated with early and distant recurrence in patients with resectable PC. Our results indicate that FR^+^ CTCs could be a promising tool to individualize treatment planning and to improve outcomes in PC. Further studies to investigate the prognostic value of CTCs detected by LT-PCR in a larger cohort are warranted.

## Data availability statement

The original contributions presented in the study are included in the article/Supplementary Material. Further inquiries can be directed to the corresponding authors.

## Ethics statement

The studies involving human participants were reviewed and approved by Ethics Committee of Nanjing Drum Tower Hospital. The patients/participants provided their written informed consent to participate in this study. Written informed consent was obtained from the individual(s) for the publication of any potentially identifiable images or data included in this article.

## Author contributions

HC, JY, and XF: study design, data acquisition, data analysis, data interpretation; HC: drafting of the manuscript; LM, XC, CL, and GL: data acquisition; YQ and WH: study concept, study design and study supervision; YQ: critical revision of the manuscript for important intellectual content; WH: contribution of CTC detection method and reagents. All authors contributed to the article and approved the submitted version.

## Funding

This study was supported by National Natural Science Foundation of China (NSFC) (No. 31971518).

## Acknowledgments

The authors thank all members of the multidisciplinary team treating pancreatic tumors at Nanjing Drum Tower Hospital for their guidance in this study. We also appreciate the technical support of Yidu Cloud (Beijing) Technology Co. Ltd., China, in extracting data.

## Conflict of interest

The authors declare that the research was conducted in the absence of any commercial or financial relationships that could be construed as a potential conflict of interest.

## Publisher’s note

All claims expressed in this article are solely those of the authors and do not necessarily represent those of their affiliated organizations, or those of the publisher, the editors and the reviewers. Any product that may be evaluated in this article, or claim that may be made by its manufacturer, is not guaranteed or endorsed by the publisher.
